# The emergence of Miocene reefs in South China Sea and its resilient adaptability under varying eustatic, climatic and oceanographic conditions

**DOI:** 10.1038/s41598-020-64119-9

**Published:** 2020-04-28

**Authors:** Manoj Mathew, Adelya Makhankova, David Menier, Benjamin Sautter, Christian Betzler, Bernard Pierson

**Affiliations:** 1 Shale Gas Research Group, Institute of Hydrocarbon Recovery, Universiti Teknologi PETRONAS, 32610 Bandar Seri Iskandar, Malaysia; 2 Department of Geosciences, Universiti Teknologi PETRONAS, 32610 Bandar Seri Iskandar, Malaysia; 3 Laboratoire Géosciences Océan (LGO), Université Bretagne Sud, 56017 Vannes, Cedex France; 4 Institute of Geology, CEN, University of Hamburg, Bundesstrasse 55, 20146 Hamburg, Germany; 5 GEO-Instituut, Campus Arenberg, Katholieke Universiteit Leuven, Celestijnenlaan 200E, B-3001 Leuven, Heverlee Belgium

**Keywords:** Stratigraphy, Geomorphology, Geophysics, Sedimentology

## Abstract

During the Miocene, extensive carbonate deposition thrived over wide latitudinal ranges in Southeast Asia despite perturbations of the global climate and thermohaline circulation that affected the Asian continent. Nevertheless, the mechanisms of its emergence, adaptability in siliciclastic-dominated margins and demise, especially in southern South China Sea (SCS), are largely speculative and remains enigmatic along with a scarcity of constraints on paleoclimatic and palaeoceanographic conditions. Here we show, through newly acquired high-resolution geophysical data and accurate stratigraphic records based on strontium isotopic dating, the evolution of these platforms from ~15.5–9.5 Ma is initially tied to tectonics and eustasy, and ultimately, after ~9.5 Ma, to changes in the global climate patterns and consequent palaeoceanographic conditions. Our results demonstrate at least two paleodeltas that provided favourable substratum of elevated sand bars, which conditioning the emergence of the buildups that inadvertently mirrored the underlying strata. We show unprecedented evidences for ocean current fluctuations linked to the intensification of the Asian summer monsoon winds resulting in the formation of drifts and moats, which extirpated the platforms through sediment removal and starvation. This work highlights the imperative role of palaeoceanography in creating favourable niches for reefal development that can be applicable to carbonate platforms elsewhere.

## Introduction

**Figure 1:**
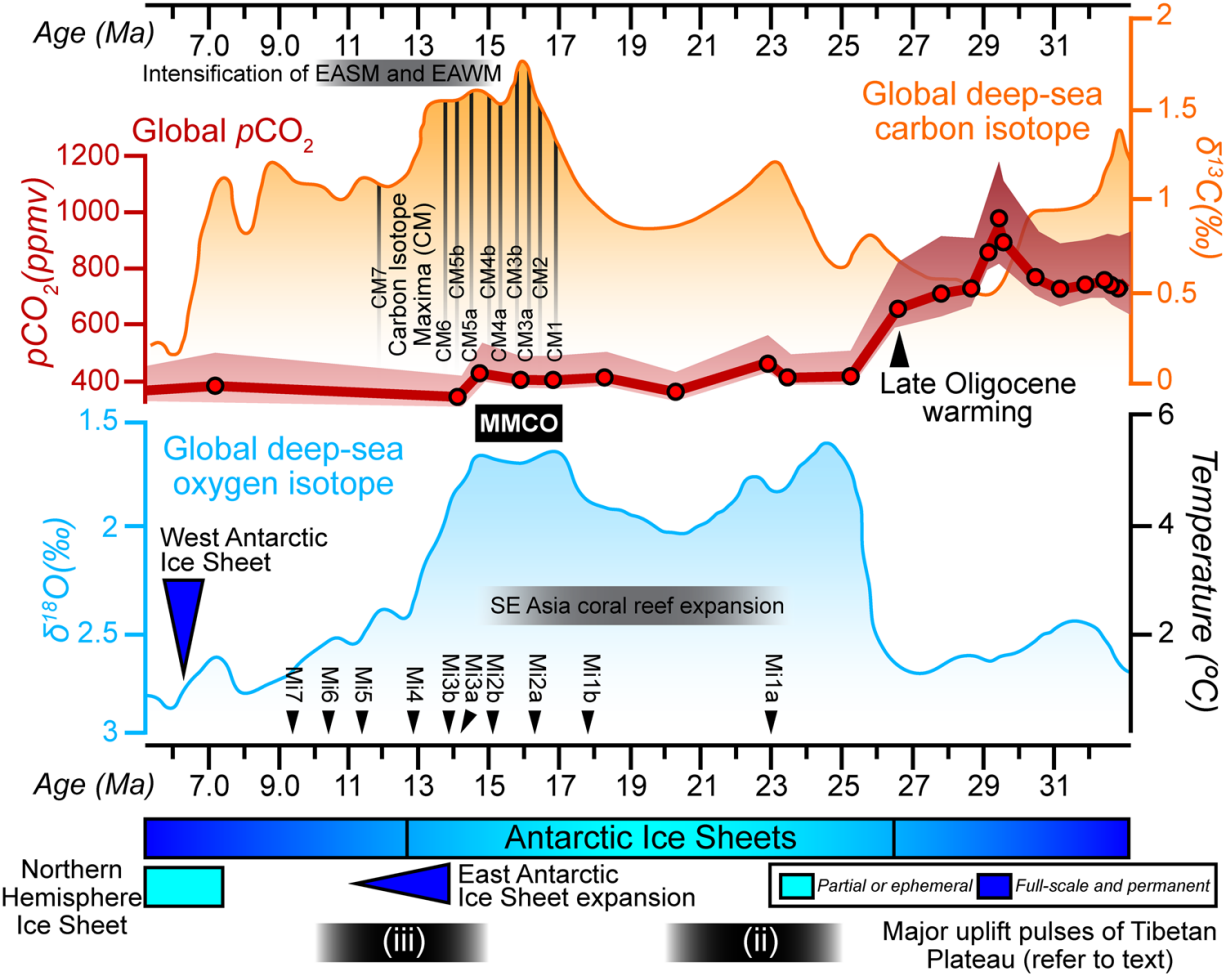
Overview of eminent climatic, atmospheric and tectonic events for the Early Oligocene to Late Miocene. Global deep-sea carbon and oxygen isotope records from 6–32 Ma are graphically depicted in orange and blue respectively. The isotope data interpreted from^10^, is an amalgam of more than 40 Deep Sea Drilling Project (DSDP) and Ocean Drilling Program (ODP) sites and demonstrates several peaks and steps that corroborate to occurrences of global warming and cooling and the expansion and decay of ice sheets. The timing of globally-recognized Middle Miocene Climate Optimum (MMCO), Late Oligocene warming, Miocene glacial events (Mi1–Mi7) and carbon maxima (CM) events based on the analysis of benthic foraminifera are interpreted from^13^. Thick red line represents the reconstruction of proxy-based partial pressure of atmospheric carbon dioxide (*p*CO_2_) that has been interpreted from^63^. Bars in light and dark shades of blue and dark blue arrows indicate the brief history of Northern Hemisphere and Antarctic ice sheets. Black bars marked with (ii) and (iii) show the temporal uplift pulses of the Tibetan Plateau (TP), which is adapted from^4^. Note that the initial uplift pulse (i) is older (ca. 40–35 Ma) than the time frame illustrated in the abscissa. Also shown are the timing of Southeast Asia coral reef expansion and intensification of the East Asian Summer Monsoon (EASM) and East Asian Winter Monsoon (EAWM) in grey bars. Figure labels were added using Adobe Illustrator version CS5.1 (http://www.adobe.com/products/illustrator.html).

A gargantuan modification of the planetary climate system and the thermohaline circulation patterns occurred in the Cenozoic with the inflection point being diffused from exceptionally critical tectonic processes that affected Asia^1,2^. At ca. 24 Ma, in close proximity to the Oligocene–Miocene boundary, the continent experienced a transformation of climatic processes from a latitudinal zonal circulation pattern to a monsoon-dominated configuration along with the evanescing of an extensively prevalent subtropical-aridity across Asia and the inception of aridity that was exclusively restricted to the hinterland of eastern Asia^3^. The Asian continent experienced a dynamic climate transition that was instigated by the Paleocene–Eocene collision of the Indian Plate with Asia and the resulting progressive uplift of the Tibetan Plateau (TP). This caused an array of feedbacks that significantly strengthened monsoon-inducing atmospheric circulation with each of its major orogenic pulses^4^ (i.e., at (i) ca. 40–35 Ma; (ii) ca. 25–20 Ma; and (iii) ca. 15–10 Ma [refer to Fig. 1 for (ii) and (iii)]). Over a span of ~30 Ma, the stepwise exhumation of the TP governed an accelerated amplification of firstly, the Indian Summer Monsoon (ISM) and the Somali Jet with the initial pulse of uplift; secondly, the Southeast Asian Monsoon, the East Asian Summer Monsoon (EASM) and the East Asian Winter Monsoon (EAWM) with 40% uplift of the TP; and lastly, the further intensification of the EASM and EAWM with 80% uplift of the TP^5,6^ (Fig. 1). This last uplift event concomitantly established the abrupt onset of the vigorous South Asian Monsoon (SAM) circulation winds at ca. 12.9 Ma^7,8^. Consequently, the gradational withdrawal and closure of the Paratethys seaway additionally strengthened atmospheric gradients and seasonal wind velocities in Asia^7^. Atmospheric circulation could have been further modified owing to changes in global ice volume (Fig. 1) and temperature drops that resulted in major global glacial events (Mi1–7) in the Miocene as documented in marine δ^18^O records^9,10^ (Fig. 1) and proxy estimates, which validated large-scale decline of CO_2_ in the Oligocene until ~24 Ma^11,12^. The buildup of organic carbon-rich sediments during the Early–Middle Miocene Climate Optimum (MMCO) augmented the inorganic carbon isotopic value of foraminiferal calcite^13^, resulting in carbon maxima (CM) events (CM1–7) as recorded by global deep-water benthic δ^13^C archives (Fig. 1). In conjunction with the aforementioned events, the opening of the South China Sea (SCS) exercised an indispensable control over climatic forcing through the exacerbation of the north–south contrast of humidity, and particularly in Southeast Asia and Sundaland, where, following a “ridge-jump” event^14^, climatic changes were triggered by the inception of seafloor spreading in the southern SCS at ca. 24 Ma^15^.

**Figure 2:**
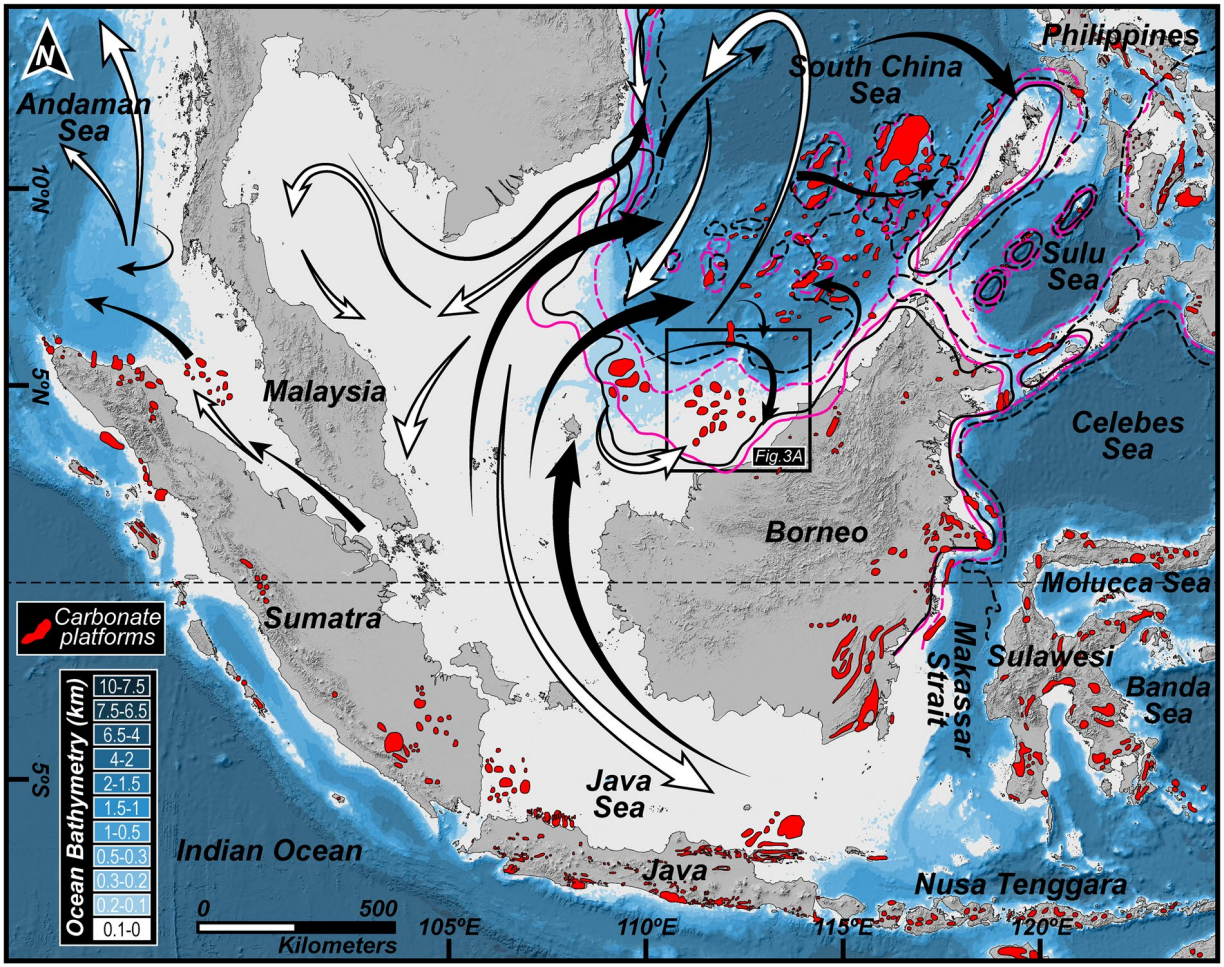
Occurrences of Cenozoic carbonates in SE Asia. Red geometries interpreted from^20^, demonstrates the distribution of Cenozoic carbonates in and around the shallow seas of Southeast Asia. Solid (50 m isobath) and dashed (200 m isobath) lines in black and pink colours that is interpreted from^45^, represent paleobathymetric reconstructions based on sea level highstands for two (2) time slices, i.e., Langhian (15 Ma) and Serravallian (12 Ma), respectively. Shades of blue show modern ocean bathymetry, while, black and white arrows indicate the general direction of present-day wind regime during summer monsoon (north-eastward) and winter monsoon (south-westward), respectively. Base map is a SRTM (Shuttle Radar Topographic Mission) Digital Elevation Model (DEM) of 30 m (1-arc second) spatial resolution (SRTM 1 Arc-Second Global elevation data courtesy of the U.S. Geological Survey, https://lta.cr.usgs.gov/SRTM1Arc) and gridded bathymetric data is from the General Bathymetric Chart of the Oceans (GEBCO) (15 Arc-Second global ocean and land terrain models courtesy of the International Hydrographic Organization [IHO] and the Intergovernmental Oceanographic Commission [IOC] of UNESCO, https://www.gebco.net/data_and_products/gridded_bathymetry_data/). The map was created using Geographic Information Systems (GIS) software ESRI ArcGIS version 10.3 (http://www.esri.com/software/arcgis/arcgis-for-desktop). Figure labels were added using Adobe Illustrator version CS5.1 (http://www.adobe.com/products/illustrator.html).

The magnitude of tectonic activity in Southeast Asia was pervasive near the Oligocene–Miocene boundary with the indentation of the Australian Plate within the southern Sunda Plate. This inherently pivotal event resulted in the tectonic restriction and withdrawal of a former deep and wide marine seaway (between 25 Ma and 22 Ma), the Indonesian Gateway, that separated the two continental shelves and culminated in widespread repercussions in terms of paleotopography, paleobathymetry, ecology and the organization of continental exposure^2^. These modifications in boundary conditions created a widespread impact to the global thermohaline circulation^16^ and to ocean gyres that previously channelled the equatorial transfer of water from the West Pacific to the East Pacific through the Indian, Tethyan and Atlantic Oceans^17^. This was synchronous with a distinct deep-sea δ^13^C excursion revealing a Late Oligocene warming^18^ (Fig. 1) and the Mi1 glaciation at ca. 23 Ma. The collisional event also created bathymetric barriers in the Philippines that restricted the deep to intermediate, nutrient-rich cool water oceanic through flow from the Pacific to the Indian Ocean, which, at ca. 24–23 Ma, is hypothesized to have facilitated the acme of Southeast Asian coral reef expansion (Fig. 1), of which 70% formed as land-attached features, following changes in trophic resources and lateral expansion of the photic zone through the emergence of newly-exposed littoral zones^19^. Despite the rarity of Paleocene and Early Eocene carbonates in entire Southeast Asia, carbonate production in the Cenozoic was observed in the shallow-marine seas of Southeast Asia^20^ (Fig. 2) with a minor progressive increment in carbonate production in the Late Eocene such as, for example, in eastern SE Asia and along the margins of marine extensional basins (e.g.^20–22^), during the Late Eocene and Early Oligocene in New Guinea (e.g.^23,24^) and in the early Late Oligocene in parts of Java and Sumatra (e.g.^25^) and the Philippines (e.g.^26^). However, at the Oligocene–Miocene boundary, there was a profound expansion in the extent of reef growth that was accompanied by a change from a larger foraminiferal to coral dominance in the shallow-water realm^27^. The acme of reefal growth in Southeast Asia (Fig. 1) lags the Oligocene coral reefs maximum of the Caribbean and Mediterranean, likely due to regional and global controls that include, but is not limited to, changes in atmospheric CO_2_ levels, *p*CO_2_ values that had fallen to pre-industrial levels (Fig. 1), oceanic Ca^2+^ and Mg/Ca ratios, and reduced salinities in humid equatorial waters.

Concurrently, in addition to all the aforesaid tectonic and climatic changes in Southeast Asia, especially in the southern South China Sea region, large deltas rapidly prograded into the surrounding deep basins at the Oligo–Miocene boundary^2,17,28^. The exact paleogeography of these Paleogene–Neogene deltas, however, is only partly known because of the limited amount of data available^29,30^ and because tectonic movements could have overprinted the depositional geometries.

**Figure 3:**
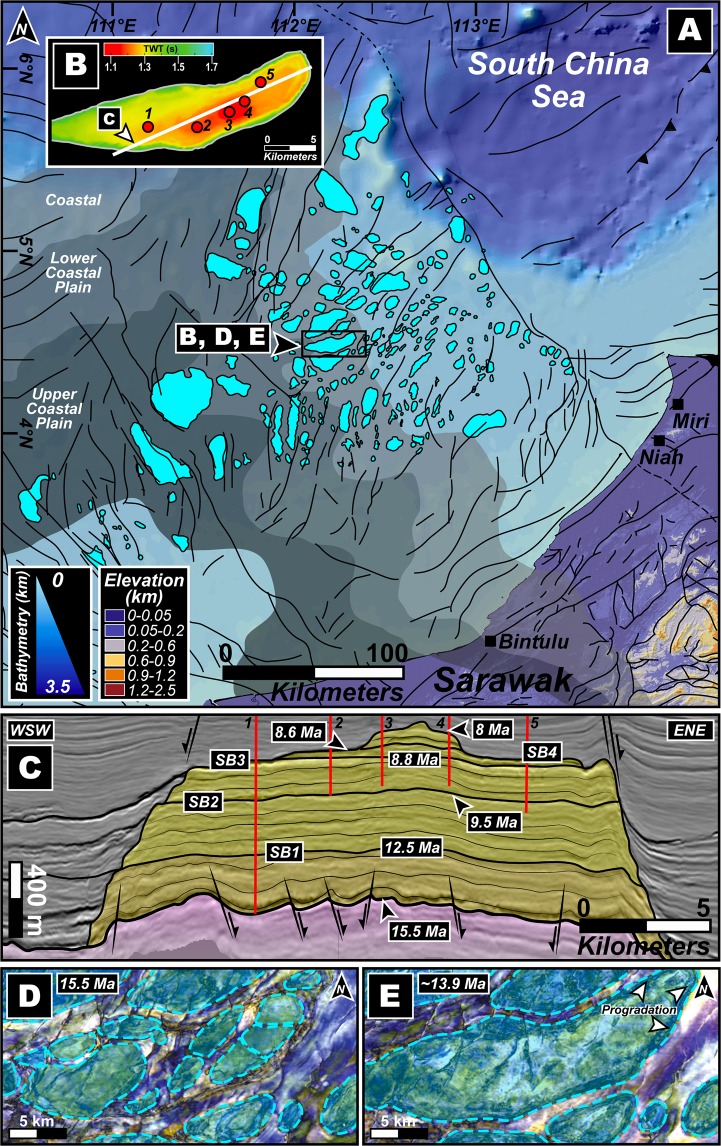
The Middle Miocene carbonate platforms of Luconia, southern South China Sea. (**A**) Bright blue geometries illustrate subsurface Miocene carbonates of Luconia spread over a spatial extent of ~4.5 × 10^4^ km^2^ identified through seismic horizon maps of the southern South China Sea and based on^31^. Note the two (2) clearly distinguishable patterns of platform growth that can be observed (i.e., linear platforms with a predominant N–S orientation toward the south and NE-SW directed platforms to the north). Color-coded continental elevation and bathymetric depth are shown along with major faults indicated with black solid lines that are based on^64^. Also shown, in shades of grey, are the modelled Miocene paleogeographic limits adapted from^29^. The paleogeography was constrained through sedimentological and stratigraphic evidences contained within clastic deposits from deep cores and onshore outcrops. Solid black bounding box shows the location and extent of the dataset used in the study (refer to methods for details of the geophysical dataset). (**B**) Top carbonate seismic horizon map of a chronologically-accordant carbonate platform that was substantially studied herein. Red circles identify locations of drilled wells that penetrated various sequences of the platform and yielded sediment cores which were interpreted in this research. White line marks the position of seismic line shown in “**C**”. (**C**) Interpreted seismic line showing the studied platform in shades of yellow and the basal substratum in pink. Also shown are stratigraphic ages and important sequence boundaries (SB) constrained through strontium isotopic dating. Drilled wells are shown in solid red lines. Faults and sense of motion is shown in black solid lines and adjacent arrows, respectively. (**D**,**E**) Spectral decomposition maps for two (2) time slices, i.e., 15.5 Ma and ~13.9 Ma. Note the individual reefal buildups in “**D**” followed by coalescing and progradation of the eastern end of the platform in a predominantly ESE and ENE direction in “**E**”. Base map in (**A**) is a SRTM (Shuttle Radar Topographic Mission) Digital Elevation Model (DEM) of 30 m (1-arc second) spatial resolution (SRTM data courtesy of the U.S. Geological Survey, https://lta.cr.usgs.gov/SRTM1Arc) and gridded bathymetric data is from the General Bathymetric Chart of the Oceans (GEBCO) (15 Arc-Second models courtesy of the International Hydrographic Organization [IHO] and the Intergovernmental Oceanographic Commission [IOC] of UNESCO, https://www.gebco.net/data_and_products/gridded_bathymetry_data/). The map was created using Geographic Information Systems (GIS) software ESRI ArcGIS version 10.3 (http://www.esri.com/software/arcgis/arcgis-for-desktop). Base maps and images in (**B**–**E**) were visualized, interpreted and constructed with Eliis PaleoScan software version 2018.1.0 (http://www.eliis.fr/products/paleoscan%E2%84%A2-software). Figure labels were added using Adobe Illustrator version CS5.1 (http://www.adobe.com/products/illustrator.html).

Reef distribution in Southeast Asia is presumed to have declined at the end of the Early Miocene owing to increased continentality, augmented oceanic ventilation and increased seasonal runoff, among others^27^. Although the deterioration was critical in the southern realms of the South China Sea, the Middle–Late Miocene Luconia platforms continued their expansion over a spatial extent^31^ of ~4.5 ×10^4^ km^2^ (Fig. 3A). However, because of a severe deficit in the availability of continuous data records of carbonates from this region, the theoretical underpinning of the mechanisms of evolution and demise of the platforms are largely speculative and prevails as a conundrum along with a dearth of tight constraints of paleoclimatic and palaeoceanographic conditions. Additionally, in this region, definite evidences of the capability of ancient reefal systems to be opportunistic colonizers by conveniently selecting a favourable siliciclastic substratum to grow on and to inadvertently mirror the morphology of the underlying bedforms that elucidate preceding morphodynamic and oceanographic processes remains shrouded in mystery. Here, informed by newly acquired high-resolution geophysical data, together with accurate stratigraphic, sedimentological and geochemical records, we show through clear evidences that the evolution of the Middle Miocene carbonate platforms in the southern South China Sea is explicitly tied initially to the response of the sedimentary system to regional geodynamics and resultant sea level oscillations, and ultimately to changes in the global climate patterns. Our data produces evidences for ocean current fluctuations and we demonstrate that one of the most critical factors governing the Late Miocene demise of the buildups was indispensably organized and controlled by the intensification of the Asian summer monsoon winds. Auxiliary to these findings, this work highlights the relevance and the imperative role of palaeoceanography in creating favourable niches and environments for reefal development that can be applicable to other Cenozoic carbonate platforms in Southeast Asia and other parts of the World.

### The emergence and adaptability of Middle Miocene carbonate systems of southern South China Sea

**Figure 4:**
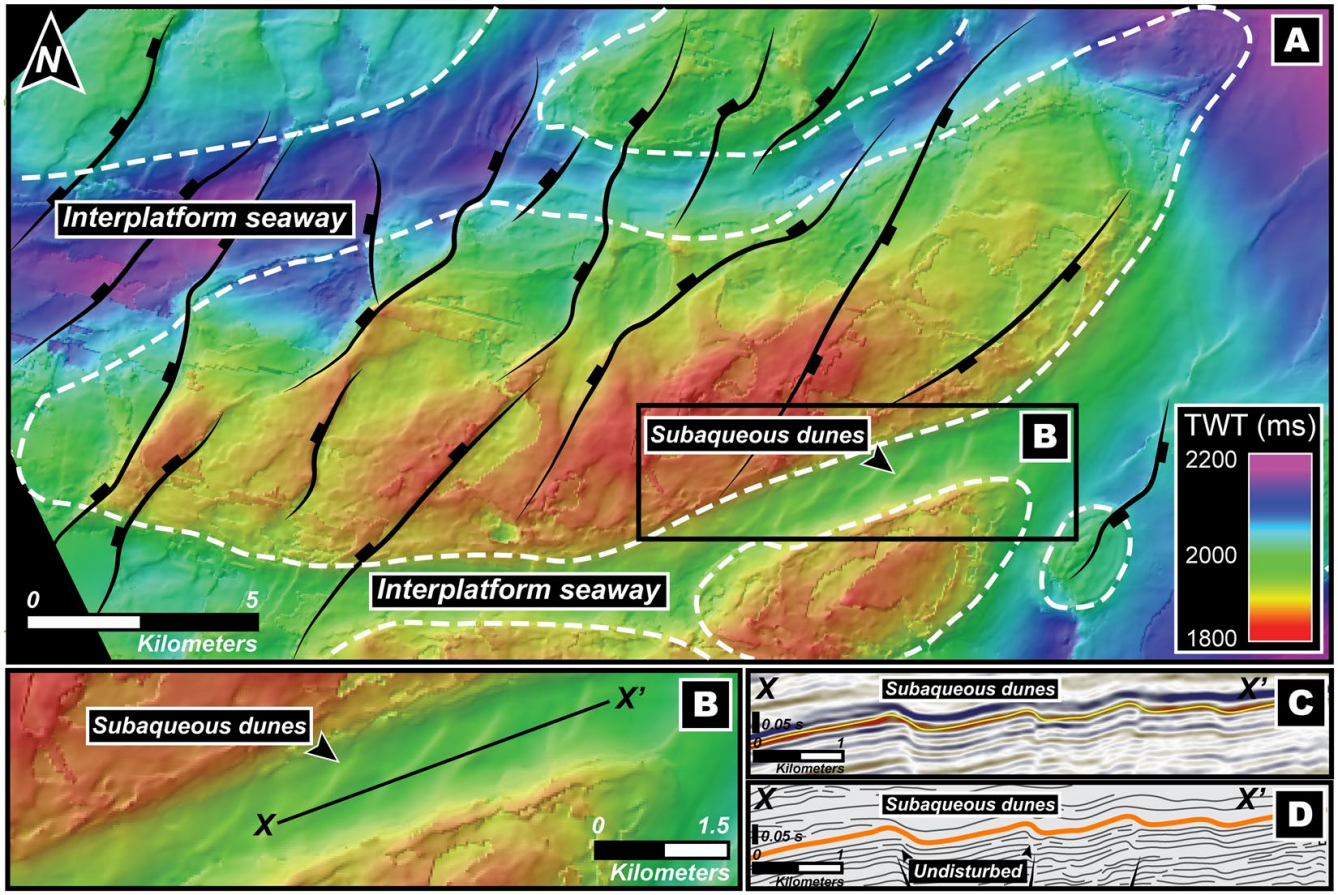
(**A**) Color-coded seismic reflection-derived horizon surface map for the time slice of ~15 Ma. White dashed-lines distinguish the spatial extent of the carbonate platforms from the interplatform seaways that host a series of subaqueous dunes. Thick black solid lines demonstrate major structural discontinuities affecting the platform and basement along with the sense of movement that resulted in an array of differentially elevated blocks. (**B**) Zoomed image of the subaqueous dunes shown in “**A**” and the black line marked X–X’ shows the position of the seismic line displayed in “**C**”. (**C**,**D**) Seismic profile across the subaqueous dunes and subsequent interpretation of the profile. Note that the subaqueous dunes are undisturbed by faults that do not penetrate the sedimentary structures. Base maps and images in (**A**–**C**) were visualized, interpreted and constructed with Eliis PaleoScan software version 2018.1.0 (http://www.eliis.fr/products/paleoscan%E2%84%A2-software). Figure labels were added using Adobe Illustrator version CS5.1 (http://www.adobe.com/products/illustrator.html).

**Figure 5:**
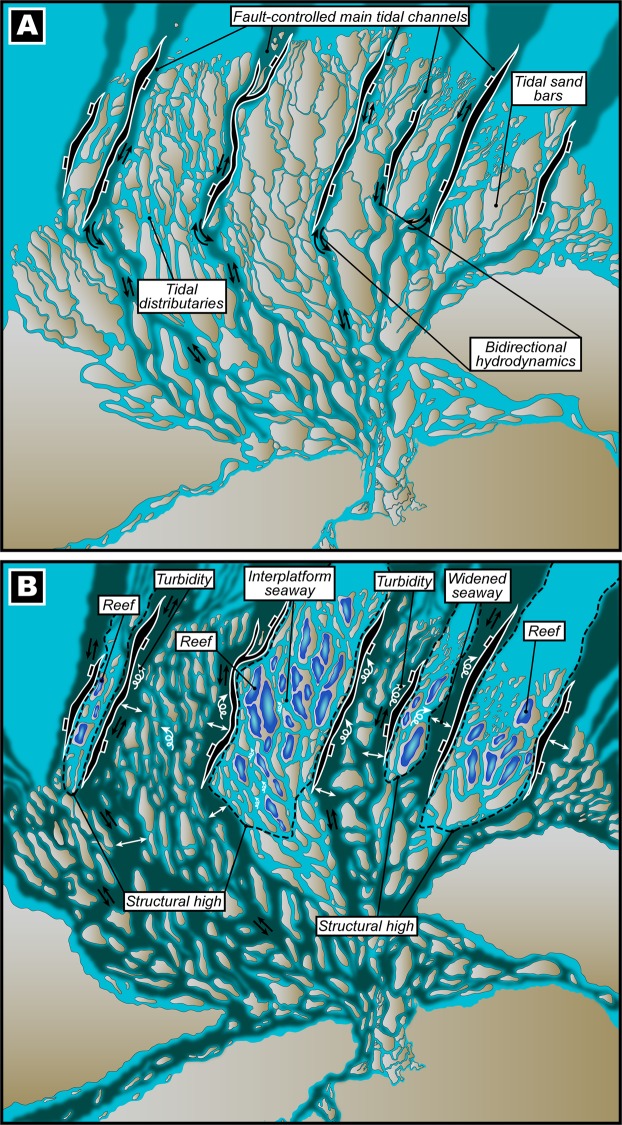
(**A**) The conceptualized evolution of a large Oligo–Miocene delta that formed under conditions of exuberant sediment supply, prolonged subsidence and accelerated sea level rise that facilitated the rapid progradation of siliciclastic material to form well-developed tidal deltaic sand bars and linear sand ridges. Regional crustal stretching and extreme thinning in the SCS^2^ resulted in wide-spread normal faulting that created flow paths for deep master channels characterized by bidirectional hydrodynamics. Smaller tidal distributaries flowed on the horsts that shaped the morphology of the sand bars and exported and redistributed siliciclastic coastal sediments within the delta system. (**B**) A further rise of eustatic levels in the Middle Miocene could have led to progressive flooding of the lowstand deltaic sand bars. Since the inundated sand bars were located on structurally-inherited elevated seafloor morphologies, the depth of the water column and photic zone would have been auspicious for biogenic ecosystems and these conditions compelled the reefal communities to establish themselves on the seaward margin of structural highs and along the margins of the tidal channels. Conversely, under rising sea levels, the structural lows became deeper and no reefs developed here and within the deep and widened interplatform seaways that could have been continually transporting continental deposits and reef debris down-slope through tidal channels contained within the interplatform seaways. However, sudden spikes of localized sediment flux could essentially flood the channels and constrict the pathways that could eventually increase tidal current velocity and turbidity. The conceptual model was constructed using Adobe Illustrator version CS5.1 (http://www.adobe.com/products/illustrator.html).

A detailed analysis of our seismic profiles and strontium isotope dating from the southern South China Sea reveals that the carbonates initiated at ~15.5 Ma (Fig. 3C,D) and it can be observed that these Middle Miocene carbonate platforms overlie distinct progradation features of paleodelta systems (see Supplementary Fig. S1), which prevailed in this location during the Oligo–Miocene transition^2,17,28^. The analysis of the base of carbonates from horizon maps and the evaluation of generic and detailed morphologies of the platforms reveals two unequivocally distinguishable patterns of platform growth (Fig. 3A). The southern set of platforms are manifested in a linear configuration with a predominant N–S orientation, contrasting with the NE-SW carbonate edifices to the north. Our seismic reflection-derived horizon surfaces, calibrated to a Middle Miocene age as constrained through strontium isotopic data, clearly displays the presence of interplatform seaways (Fig. 4A) containing subaqueous dunes (Fig. 4A–D). Additionally, the interplatform seaways that are confined between the carbonate platforms display an internal architecture that demonstrates repeated incisions by possibly tidal channels and filling phases from above the 15.5 Ma boundary (see Supplementary Fig. S1) because interplatform seaways predominantly represent passages of amplified tidal current velocity^32,33^. Thus, based on our evidences, the interplatform seaways in our data can be interpreted as corridors characterized by tide-driven hydrodynamics that may have formed the large subaqueous dunes. The subaqueous dunes appear to more or less follow an orientation that is akin to the regional structural trend, which highlights the fact that the tectonic history of an area can often be inherited and control the location of subsequent geomorphic features; however, from our data, we observe that while the paleotopography could have laid the foundation for initial sediment accretion, most of the subaqueous dunes appear to be undisturbed and devoid of faulting (Fig. 4C,D). Below the 15.5 Ma boundary, observations can be made of a sedimentary architecture resembling a progradational deltaic system with numerous channels incepted during progradation and that operated as networks to facilitate the transport of sediments from continental land masses to deep marine basins. The causative mechanisms of overlying carbonate development within a deltaic setting in this area are envisaged through a conceptual model (Fig. 5) that is presented herein. During the Oligo–Miocene transitional period, prolonged subsidence and extensional normal fault systems were ubiquitous in the southern Sundaland following regional crustal stretching and extreme thinning in the SCS^2^. Because the balance between deposition and erosion is predominantly influenced by hydraulic velocity and sediment flux^34^, under moderate velocities and exuberant sediment supply, as in the case of the present study location (e.g.,^29,30^), along with the subsequently created accommodation space and the onset of rapid sea level rise could have resulted in the progradation of siliciclastic material along coastlines in the form of deltas with developed sand bars and linear sand ridges (Fig. 5A). Furthermore, the subsequently formed structural dislocations would have been favourable niches for bi-directional tidal channels and the smaller tidal distributaries (Fig. 5A) could have fed the master channels and aided in the export and redistribution of siliciclastic sediments. Until the Middle Miocene, owing to further escalated eustatic levels, successive flooding of the lowstand deltaic features such as tidal sand bars could have provided an auspicious period for reefal communities to establish on the seaward margin of structural highs and atop strata hosted by the older deltaic siliciclastic deposits that could be maintained on the structural highs (Fig. 5B); thus, mimicking the geometry of the substratal deltaic sand bodies. This growth pattern can be witnessed in modern analogs of other tropical mixed siliciclastic-carbonate systems around the world and has been substantially documented in^35^. The reefal communities would have been compelled to opportunely chose the inundated tidal sand bars as substratum that could have been located on structurally-inherited, elevated seafloor morphologies because the height of the water column and photic zone would have been optimal for biogenic ecosystems to flourish. As seen in our results, colonization does not appear to exist in most of the interplatform seaways (see Supplementary Fig. S1) that was created following a transition from a deltaic setting to a marine system. This could illustrate that continental deposits and reef debris could have been constantly exported down-slope along possible tidal channels contained within these interplatform seaways that could resemble present-day river network patterns as mentioned in^31^. Because the sediment load transferred by the channels is regulated by the influence of continental landscape dynamics that are orchestrated by endogenic and exogenic forcing, transiently conveyed sudden spikes of localized sediment flux can inundate the channels and constrict the pathways that eventually incite an exacerbation of tidal current velocity and turbidity (Fig. 5B).

### Late miocene shift in carbonate deposition dynamics

The copious accumulation of Mid–Late Miocene carbonate platforms in southern South China Sea transpired following regional tectonic events that occurred during the Oligocene to Middle Miocene and involved a network of deep-rooted normal faults^36^ associated with continuous subsidence. The ensuing basement geometry was characterized by differentially elevated blocks that could have contained tidal sand bars atop which carbonate platforms grew during the intermediate Miocene and many of these locations are still presently sites for reefal edifices^37^.

The modern oceanic currents in this region are principally governed by a dynamic wind gradient from the Pacific to the Indian Ocean and the Southeast Asian and South Asian Monsoon^38^. These semi-annually reversing winds that cause surface current patterns to reciprocally reverse are generally directed north-eastward during the summer monsoon and south-westward during the winter monsoon (Fig. 2), and are integral in the hydrological, chemical, sedimentological and sea-surface circulation dynamics in this part of the planet. Indeed, counter-clockwise eddies and stronger currents are additionally observed in the western part of the South China Sea^36^.

**Figure 6:**
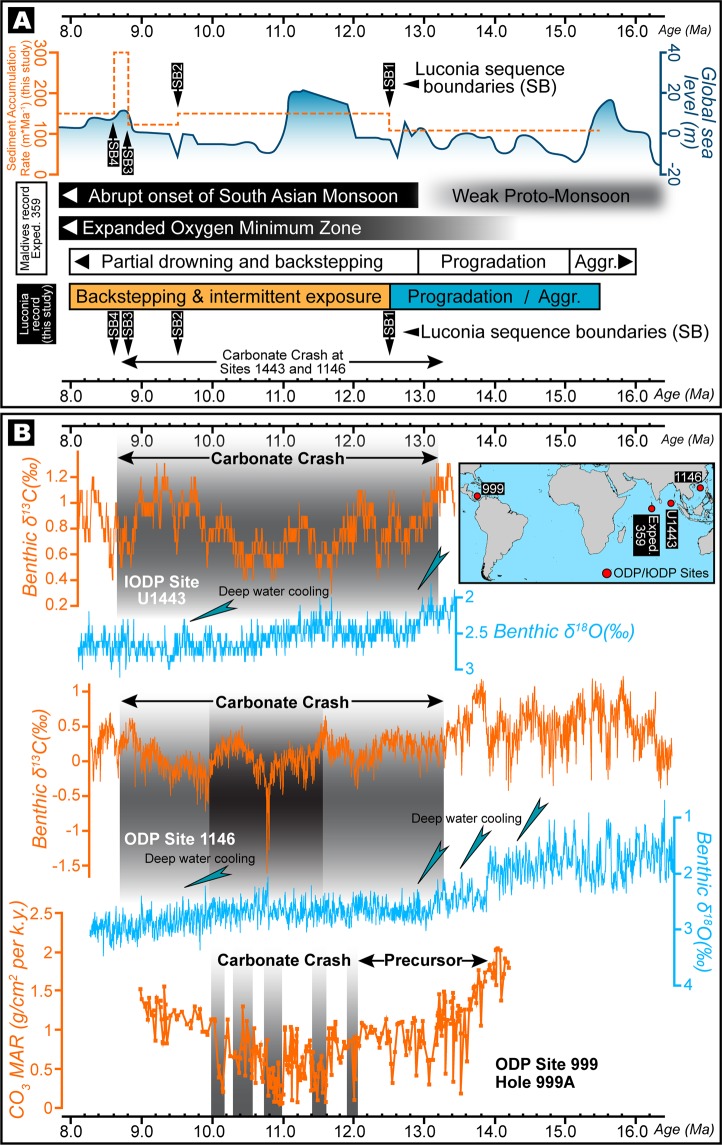
(**A**) Regime diagram showing the age of sequence boundaries (SB1–4), important stratigraphic breaks and changes in platform evolution proposed in this work and sedimentation rate of the studied Luconia platform. Also shown is the growth cycles of carbonate edifices in the Maldives adapted from^7^, since the Middle Miocene along with timing of major climatic and oceanographic phenomena observed in the Maldives record. Global sea level is graphically represented in blue and is interpreted from^65^. (**B**) Middle to Late Miocene (16–8 Ma) benthic foraminiferal δ^18^O and δ^13^C records from IODP Site U1443 (Ninetyeast Ridge, Indian Ocean) and ODP Site 1146 (northern South China Sea), and carbonate mass accumulation rate (CO_3_ MAR) for ODP Site 999, Hole 999 A (Colombian Basin, Caribbean) compiled and interpreted from^49,51,66^ respectively. Grey shaded bands indicate timing of “Carbonate Crash” event that marks episodes of intense impoverishment in carbonate accumulation and strong carbonate-dissolution. Teal coloured arrows show main phases of glacial expansion/deep water cooling. Figure labels were added using Adobe Illustrator version CS5.1 (http://www.adobe.com/products/illustrator.html). The inset map shown in (**B**) was created using Geographic Information Systems (GIS) software ESRI ArcGIS version 10.3 (http://www.esri.com/software/arcgis/arcgis-for-desktop).

**Figure 7:**
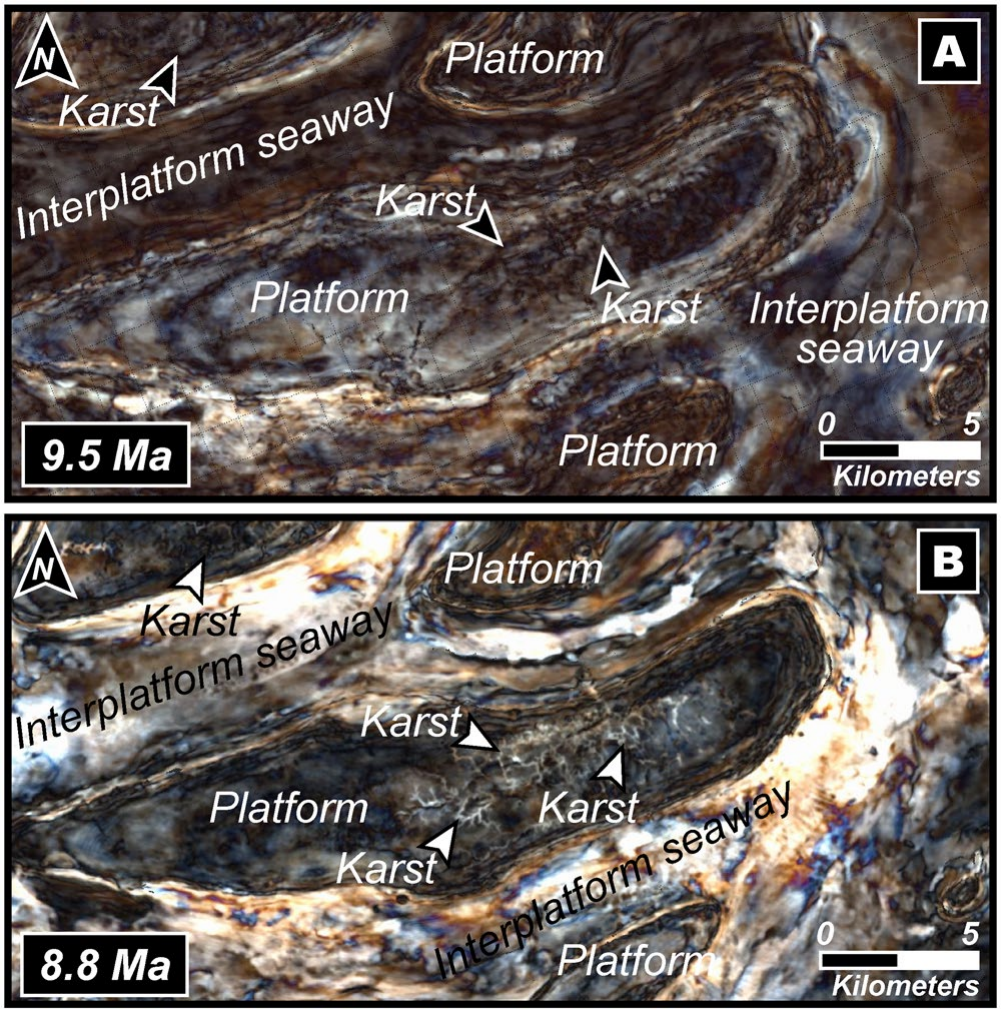
(**A**,**B**) Spectral decomposition maps for two (2) time slices, i.e., ~9.5 Ma and ~8.8 Ma showing karsts as a result of a lowering of global sea levels and exposure of the platform. Base maps in (**A**,**B**) were visualized, interpreted and constructed with Eliis PaleoScan software version 2018.1.0 (http://www.eliis.fr/products/paleoscan%E2%84%A2-software). Figure labels were added using Adobe Illustrator version CS5.1 (http://www.adobe.com/products/illustrator.html).

**Figure 8:**
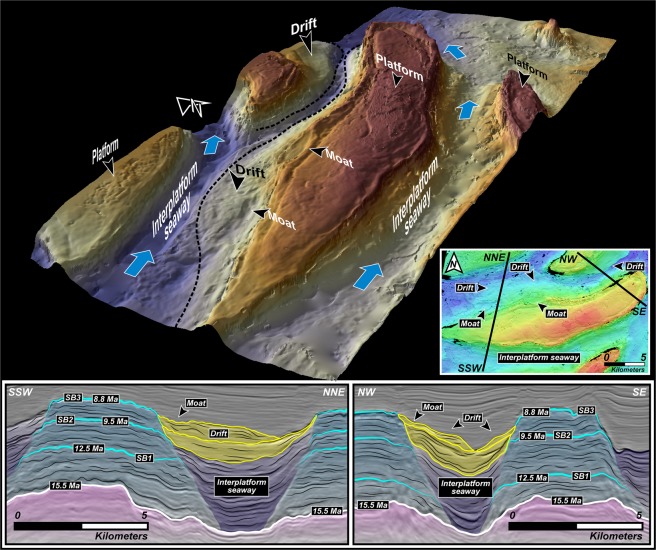
Oceanic current controlled contourites in the southern South China Sea. 3D view of the horizon pertaining to ~8.8 Ma showing intense Asian summer monsoon wind-driven current deposits identified in this study with well-defined drift and moat geometries surrounding the carbonate platforms in Luconia. Also shown are seismic cross-sections of the drift bodies and moat features along with ages of platform sequence boundaries (SB) recognized and interpreted herein. Base maps and images were visualized, interpreted and constructed with Eliis PaleoScan software version 2018.1.0 (http://www.eliis.fr/products/paleoscan%E2%84%A2-software). Figure labels were added using Adobe Illustrator version CS5.1 (http://www.adobe.com/products/illustrator.html).

**Figure 9:**
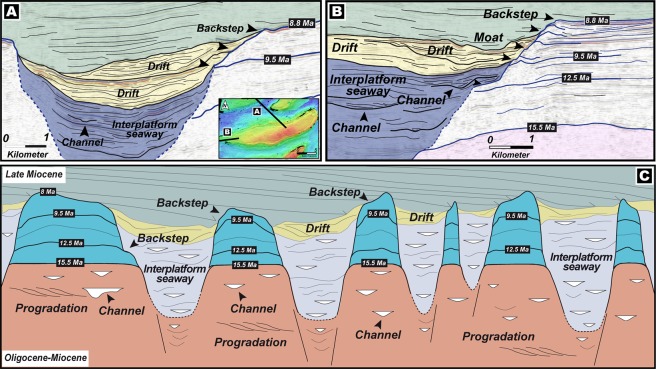
(**A**,**B**) Interpreted seismic line showing drift bodies and moat geometries and backstepping of the platform related to sea level fluctuations and contourite deposition following strong currents from the abrupt intensification of the Asian summer monsoon winds after ~9.5 Ma. (**C**) Conceptual sketch summarizing the stratigraphy of the study area (refer to text for details). Base maps and images in (**A**,**B**) were visualized, interpreted and constructed with Eliis PaleoScan software version 2018.1.0 (http://www.eliis.fr/products/paleoscan%E2%84%A2-software). Figure labels were added using Adobe Illustrator version CS5.1 (http://www.adobe.com/products/illustrator.html). The conceptual sketch in (**C**) was constructed using Adobe Illustrator version CS5.1 (http://www.adobe.com/products/illustrator.html).

High-resolution geophysical data obtained and interpreted by us in this work and augmented by detailed core and thin section descriptions shown in^39^, and strontium isotopic chronology that was integrated and calibrated with palaeontological records, facilitated the robust constraining of the age and limits of sequence boundaries (SB) (Figs. 3C and 6A) with carbonate packages that reflect distinct evolutionary characteristics evidencing the adaptability of corals to successive periods of climatic and tectonic stress. Because all the carbonate buildups of this region are stratigraphically well-constrained and documented to be of Middle–Late Miocene age^15^, we analysed an ideally representative and chronologically-accordant carbonate platform in this region (Fig. 3B,C) that demonstrates a phase of aggradation and progradation during the inception stage (Fig. 6A). This phase can be associated with global climatic transitions and regional tectonics-induced eustatic fluctuations that disrupted platform development during events of lowstand. Our interpretation of the carbonate system, chronology and fault architecture of the southern South China Sea suggests that the platform initiated at ~15.5 Ma (Fig. 3C,D), in the later stages of the Middle Miocene Climate Optimum (MMCO) during which the global eustatic levels were considerably high (Fig. 6A), compelling the platform to aggrade on deltaic sand bodies localized on elevated inverted sedimentary substratum (Fig. 3C and S1). Following the aggradation phase, the carbonate banks that reveal syn-depositional faulting, expanded laterally for ~2 km through progradation in ESE and ENE directions (Fig. 3E) that is a diagnostic feature of platform response under lowered sea levels. Hence, we associate this expansion with the transition of the planetary climate system from the warm MMCO towards a cooler setting accompanied by a global eustatic drop during the Mi3b glaciation event owing to the proliferation of the East Antarctic Ice Sheet at ~13.9 Ma (Figs. 1 and 6A). This period of platform extension lasted until ~12.5 Ma marked by the sequence boundary SB1 (Fig. 6A) where the carbonate platform portrays a distinct change in evolutionary pattern and appears to shrink through backstepping/aggradation as global sea levels climbed (Fig. 6A). There was exposure of the platform at ~9.5 Ma that is reflected in the global eustatic curve as a steep drop in sea level (Fig. 6A), and this coincides with features resembling karsts (Fig. 7A) represented by intensively dissolved and leached limestone in the upper parts of the seismic unit in the cored intervals as described in^39^, hence characterizing subaerial exposure and represents the SB2 of the edifice. At ~8.8 Ma, as global sea levels rose once again, the carbonates continued a similar pattern of backstepping/aggradation as seen in the preceding sequence till SB3 (Fig. 6A) along with dissolution features (Fig. 7B) that could be related to local tectonic events^40,41^ promoting sporadic exposure. It is interesting to note that the sediment accumulation rates (Fig. 6A) calculated for the platform demonstrates a relatively stable trend of slow growth and devoid of sharp peaks before the start of the next seismic sequence. A dramatic spike in accumulation rate is seen from ~8.8 Ma that terminates with the SB4 at ~8.6 Ma (Fig. 6A). During this period, the platform appears to shrink drastically through a pronounced backstepping/aggradation of ~13.2 km in an ENE direction. While a perceptible rise in global eustatic levels are identified after ~9.5 Ma and ~8.8 Ma, large-scale lobate clinoform features are explicitly recognizable in our seismic sections and horizon map that we interpret as contourites with well-defined drift and moat geometries (Figs. 8, 9A,B), evidencing a dynamic deposition by intense wind-driven currents showing an unambiguous physical record of the strong influence of monsoonal winds. With the subsequent rise of eustatic sea levels, strong currents could have scoured large parts of the Luconia platforms that may have prevented the regeneration of shallow-marine ecosystems. Along with rising sea levels, this could have been one of the critical factors that forced the platform to backstep over a large distance (Fig. 3C) until its demise at ~8 Ma. This phenomenon marked an eminent change in depositional dynamics from a system influenced by global sea level fluctuations to an intrinsically organized framework controlled by wind-driven oceanic currents.

### Discussion and implications

Conclusive inferences obtained from our results and interpretation of the same suggests that the palaeoenvironment of the southern South China Sea was initially controlled by regional tectonic activity and eustasy in the Early Miocene that saw the widespread progradation of deltas into deeper marine basins^2,17,28^. While the existence of some paleodeltas are documented because they are conformant with present-day shorelines (e.g., Rajang paleodelta^42^), many remain elusive owing to an absence of accordant current shorelines, burial by overlying sediments and morphodynamic processes over geological time. The morphodynamic and oceanographic processes that influenced these deltas are much less understood, peculiarly, due to a lack of direct supportive evidences and uncertainties regarding paleoclimates and paleotopography. A few studies have focused on the sedimentological aspects of prominent paleodeltas in this region using well data, outcrop descriptions that could be considered as equivalents to offshore geology and by considering regional models (e.g.^30^). Our findings confirm and precisely highlight the presence and classification of at least two paleodeltas by observing the growth patterns of Middle Miocene carbonate platforms that we demonstrated to have evolved on a substratum organized by deltaic sand bodies and during growth, inadvertently mimicked the underlying strata (Fig. 5B). The horizon map illustrating the base of carbonates in the Luconia zone of southern South China Sea clearly shows two distinct growth morphologies/geometries that are predominantly oriented N–S in the southern platforms and NE–SW in the northern platforms, respectively (Fig. 3A). The elongated morphologies of the carbonate platforms located in the south are indicative of developing on wave-dominated delta-derived sand bars. The direction of the paleoshoreline hosting the delta could have trended roughly in an E–W direction that is perpendicular to the sand bars and is consistent with the modern coastline of the north-western region of Borneo Island (Figs. 2 and 3A). On the other hand, the platforms in the north portray edifices that are sizeably wider and thicker with definitive appearances of interplatform seaways (Figs. 3A and 4A) that can be traced to the deeper zones in our seismic profiles (see Supplementary Fig. S1). Inferences regarding the origin of the sand bars (Fig. 5A) chosen by the northern platforms to establish themselves on suggest that they were deposited by a tide-dominated delta system and the presence of extensive laterally-channelized sequences in the Oligocene–Miocene sediments below the ~15.5 Ma boundary, as visualized from our seismic profiles (see Supplementary Fig. S1), further validates our interpretation. The paleoshoreline could have been oriented in a trend that is perpendicular to the general NE–SW direction of the platforms, which is congruent to previous estimations of a NW–SE direction of ancient coastlines in this area with the offshore basins deepening towards the north and NE (e.g.^30,43,44^). Modelled paleogeographic locales of the coastal plain (Fig. 3A) (e.g.^29^), changes in subsurface sedimentology of clastic sequences that predate the carbonate platforms (e.g.^30^) and paleobathymetries (Fig. 2) (e.g.^45^), provide additional confirmation for the paleoenvironmental setting that is discussed in this work.

Considering the immense volume of basin-filling clastic sediments since Oligocene–Early Miocene from the hinterland of the coastlines that are perpendicular and adjacent to each other, with a NW–SE trend in the western side and E–W trend in the southern side of Borneo, it can be deduced that the flux would have to be sourced from uplifted landscapes containing monumentally high relief. These imposing orogens could have guised as obstructions that sheltered the southern South China Sea, and as a result, considerably altered wind, oceanographic, morphodynamic and depositional patterns. As mentioned previously, our results based on seismic reflections, core data and supplemented by precise isotopic chronology of Luconia carbonate platforms displays a good correlation with regional tectonic events and global sea level evolution in the Middle Miocene; however, in the Late Miocene, a strong shift towards planetary climate system as the primary controlling factor is evidently observable. The evolutionary pattern of the platform, which initiated at ~15.5 Ma, bears close resemblance to the growth cycles of carbonate edifices in the Maldives^7^ since the Middle Miocene, in terms of timing of aggradation/progradation, intermittent drowning and platform backstepping (Fig. 6A). While the initial stages of aggradation reflected in our data can be validated through a global eustatic high of the MMCO (Figs. 1 and 6A), platform progradation to the ENE and ESE (Fig. 3E) can be attributed to a wind regime that was generally directed to the eastern side of the platforms, because carbonates favourably tend to prograde towards the leeward side^7,46^. This wind pattern is peculiar of the Asian summer monsoon winds that control oceanic current systems and akin to the present-day climatic circulation experienced in the entire South China Sea^38^ (Fig. 2). Although the summer monsoon winds affected this region in the Middle Miocene to Late Miocene, its effects during platform inception could have been weaker, and we base this postulation on two lines of evidences. Firstly, the aforementioned high relief topographic barriers that surrounded the area could impede on-coming winds from the west, and secondly, the absence of contourite deposits driven by wind-controlled currents in the deeper sequences from ~15.5 Ma to ~9.5 Ma. During the later Late Miocene, a sudden initiation of oceanic current controlled sedimentation surrounding the platforms in the form of drift and moat geometries are depicted in the horizon map constructed herein (Figs. 8, 9A and 9B). Currents have the capability to alter the morphology of platform slopes through sediment removal and starvation, by generating drifts around carbonate edifices that in seismic profiles appear as convex outward prograding features which onlap and bury sequences of platform carbonates, by forming moats at the toe of slopes. The application of such a stress on reefal systems can result in backstepping of the bank margins or slope steepening^7,47^. We propose that the drift and moat formation in parts of the southern South China Sea started after ~9.5 Ma (Figs. 8, 9A and 9B) when the platforms backstepped in an ENE direction (Fig. 9B) following morphological modification of the carbonates that could more than likely have been orchestrated by intense currents generated by the amplification of the Asian summer monsoon winds along with rising sea levels, resulting in the shrinking of the buildups. Carbonate platforms in the Maldives recorded similar drift bodies and moat features with the sudden onset of intense Asian summer monsoon winds at ~12.9 Ma and a predated weaker proto-monsoon^7,8,47^ (Fig. 6A) and this would imply a considerable time lag in the onset of strong summer monsoonal winds in southern South China Sea that could be dictated by the highly elevated orogenic barrier. Though we cannot completely disregard the probable existence of a weaker current system that was incapable of creating drift sequences prior to later Late Miocene, the abrupt intensification of the northeast-blowing summer monsoon winds, strong currents and subsequent leeward backstepping after ~9.5 Ma is likely to be due to a lowering of topographic elevation of the western orogens that previously protected the area. Indeed, previously documented paleogeographic reconstructions have testified that the western orogen no longer separated the southwestern and southern South China Sea in the later Late Miocene^45^. We present a conceptual sketch (Fig. 9C) summarizing the stratigraphy of the study area. The figure illustrates carbonate growth atop Oligocene–Early Miocene deltaic tidal sand bars that were located on structural highs and the presence of interplatform seaways that formed in response to rising eustatic conditions and remained confined in between the growing platforms. The model demonstrates the major backstepping events related to sea level fluctuations and contourite deposition as a result of strong currents from the abrupt intensification of the Asian summer monsoon winds after ~9.5 Ma that formed well-defined drift bodies and moats.

We expand upon our data and demonstrate that carbonate sediment accumulation rates show generally lowered sedimentation from ~15.5–8.8 Ma followed by a sudden spike after ~8.8 Ma to ~8.6 Ma, following which, the rates recede until the demise of the platform at ~8 Ma (Fig. 6A). Despite having only a few global eustatic highstands and Miocene glaciation events (Mi2b–Mi7) in the Middle–Late Miocene (Fig. 1), a prolonged episode of reduced carbonate deposition has been witnessed globally between ~13.2 Ma and ~8.7 Ma. This marked a decline and an intense impoverishment in carbonate accumulation and strong carbonate-dissolution episodes termed “Carbonate Crash”, that was initially observed in the tropical eastern Pacific Ocean at the ODP site 846^48^, and later at ODP sites 999 and 1146 in the Caribbean Sea^49^ and northern South China Sea^50^, respectively, and more recently at IODP site U1443 in the equatorial Indian Ocean^51^ (Fig. 6B). One of the fundamental reasons for the Carbonate Crash was interpreted to be a feedback to changes in deep-water circulation and shoaling of the carbonate compensation depth (CCD) and the crash event is essentially followed by a “Biogenic Bloom” that is an epitome of a major increase in biogenous production and accumulation^52^. While these phenomena are better constrained through specialized geochemical analysis, from our results, we posit that after SB1 (Fig. 6A), the low carbonate sediment accumulation rates reflected in our data from the southern South China Sea could likely be linked to the “Carbonate Crash” because it is coeval with the crash in northern South China Sea at ODP site 1146 (Fig. 6B). Moreover, according to the globally accepted cognizance of this event, a “Biogenic Bloom” always follows the crash^49^; thus, we centre our inference on the fact that the timing of the end of the crash at ODP site 1146 and IODP site U1443 is synchronous with the large spike in sediment accumulation rates in Luconia after ~8.8 Ma (Fig. 6). Nevertheless, this bloom was short-lived in the southern South China Sea due to the abrupt onset of the intensified Asian summer monsoon and accompanying vigorous currents that played a vital role in the extirpation of the carbonate buildups. However, an interesting possibility for the demise of carbonate buildups that cannot be overlooked is that a sudden abnormal surge in nutrient levels and increased productivity resulting in higher carbonate mass accumulation rates can lead to the drowning of megabanks as observed in the Northern Nicaragua Rise (e.g.^53^). While it is recognized that several factors including, but not limited to, intensity of chemical weathering, changes in large-scale thermohaline circulation patterns, closure of important seaways and increased continentalization can combinedly contribute to carbonate deterioration, our work provides convincing evidences for confirming, i) the evolutionary mechanism of carbonates under differential tectonics in the southern South China Sea; ii) the adaptability of biogenic ecosystems to resiliently establish themselves on unfavourable substratum under adverse eustatic fluctuations, high turbidity and constant clastic influx in this region; and iii) one of the crucial, if not the most crucial, factors governing the demise of carbonate platforms in southern South China Sea through the indispensable domination of monsoonal wind-driven oceanic currents that is regulated by planetary climatic circulation.

## Methods

### Geophysical data and interpretation

The interpretation of carbonate sequences and related morpho-structures was supported by a combination of high-resolution 3D seismic profiles calibrated with well and core data. A world-class seismic dataset covering an area of 420 km^2^ with a bin grid of 12.5 m x 12.5 m and sampling rate of 4 ms was fully integrated in our regional study. The dataset that includes 1016 inlines and 1357 crosslines with a 25 m step were interpreted using Eliis PaleoScan software. The data are characterized by normal SEG polarity, where an increase of acoustic impedance is shown in positive amplitude, and a dominant frequency of 30 Hz within the platform. Applying an average velocity range of 1965–2547 ms^−1^, the maximum vertical resolution was identified as 16–22 m.

Thirteen horizons from bottom to top were picked based on the occurrence of acoustic impedance peaks, using the methodology by^54^. The seismic cross-sections constructed therein were vertically exaggerated (10×) to optimize the visualization of carbonate platform morpho-structures.

The outstanding quality of our 3D seismic cube allowed to recognise detailed sedimentary and structural features such as intra-sequences faults, karsts, channels and reefs among others, with the aid of multiple seismic attributes. Spectral decomposition was identified as the most appropriate and functional attribute to convey karst pattern and reef rim. For reef identification two frequency combinations were used; i) 18/32/60 using Short Time Fourier Transform (STFT), and ii) 23/25/30 Continuous Wavelet Transform with Morlet wavelet (M). Karst pattern were highlighted with a combination of 9/32/60 Hz (STFT) and 15/18/23 Hz (M). Resulting delineations of reef and karst features were calibrated with core data.

The time-depth relationship, which was used to measure the thicknesses of the carbonate sequences in each growth phase as well as identify the accurate fault dip and talus angles, was constructed using three check shots with velocity values to create a brief velocity model for depth cube. The presence of a gas chimney in the middle of the platform represents a processing artefact that could not be reduced in the current study.

### Strontium isotopic chronology

Two (2) chemostratigraphic analyses in Luconia were conducted in previous studies by^55^ of Shell Sarawak and the Commonwealth Scientific and Industrial Research Organisation (CSIRO), Australia, in 2009 that were compiled and integrated by us. The latest chronological dataset that was produced herein involves the measurement of Sr isotope constitution in a VG 354 thermal ionization mass spectrometer at the CSIRO radiogenic isotope facility at North Ryde, Australia. The ^87^Sr/^86^Sr ratios were denominated as the average of 54 measurements of ion intensities and normalized to ^86^Sr/^88^Sr = 0.1194 using an exponential correction law. During the course of measurements, strontium standard reference NBS987 resulted in ^87^Sr/^86^Sr values of 0.710235 (2σD error deviation, n = 54). Numerical ages were derived from the look-up table of^56,57^, and from the curves of^58,59^. The earlier study contains an extensive dataset of ^87^Sr/^86^Sr values by^59^ and the isotope analysis was conducted on 137 samples. All ^87^Sr/^86^Sr data were adjusted to a certified NBS987 value of 0.710248. Both ^87^Sr/^86^Sr ratios and age calculations correlate very closely. The obtained data were then integrated with palaeontological records based on the identification of larger foraminifera.

### Sediment accumulation rate

Accumulation rates were computed in meters per million years (m.Ma^−1^) as this ratio is equivalent to the Bubnoff unit^60^. The accumulation rates were estimated from age-depth plots by drawing best-fit lines over successive depth intervals from seismic data. The age-depth relationship was established based on biostratigraphic and strontium isotopic ages from core data over averaged thicknesses for each seismic unit. Compaction adjustment has not been accounted for, due to the absence of core data within the siliciclastic overburden, thus, they represent minimal values. It must be noted that the rates are from the vertical dimension, which indicates that they represent only a part of the clinothem accumulation and lateral progradation rates is estimated to be >100 times greater than vertical accumulation^61^.

### Core description and sedimentary facies

To characterize the evolution of the carbonate platform, a detailed description of five (5) wells (Fig. 3B,C) that includes 378 m of core was performed (please refer to^39^) in terms of lithological changes using 10% HCl, component composition and their abundance, grain size, texture using Dunham classification^62^, diagenetic overprints, visual porosity and pore types. Sedimentological dataset was used for identification of litho-facies types.

## Supplementary information

Supplementary information.

## Data Availability

The datasets generated during and/or analysed during the current study are not publicly available due to confidentiality regulations set by Petroliam Nasional Berhad (PETRONAS), Malaysia. However, data are available from the authors upon reasonable request and with permission of Petroliam Nasional Berhad (PETRONAS), Malaysia.
