# Author Correction: The emergence of Miocene reefs in South China Sea and its resilient adaptability under varying eustatic, climatic and oceanographic conditions

**DOI:** 10.1038/s41598-020-66550-4

**Published:** 2020-06-04

**Authors:** Manoj Mathew, Adelya Makhankova, David Menier, Benjamin Sautter, Christian Betzler, Bernard Pierson

**Affiliations:** 1 Shale Gas Research Group, Institute of Hydrocarbon Recovery, Universiti Teknologi Petronas, 32610 Bandar Seri Iskandar, Malaysia; 2 Department of Geosciences, Universiti Teknologi PETRONAS, 32610 Bandar Seri Iskandar, Malaysia; 3 Laboratoire Géosciences Océan (LGO), Université Bretagne Sud, 56017 Vannes, Cedex France; 4 Institute of Geology, CEN, University of Hamburg, Bundesstrasse 55, 20146 Hamburg, Germany; 5 GEO-Instituut, Campus Arenberg, Katholieke Universiteit Leuven, Celestijnenlaan 200E, B-3001 Leuven, Heverlee Belgium

Correction to: *Scientific Reports* 10.1038/s41598-020-64119-9, published online 28 April 2020

The Acknowledgements section in this Article is incomplete.

“A.M., M.M. and B.S. gratefully acknowledge Dr Michael Poppelreiter for facilitating access to geophysical data for research purposes and for acquiring permission to publish the same.”

should read:

“A.M., M.M. and B.S. gratefully acknowledge Dr Michael Poppelreiter for facilitating access to geophysical data for research purposes and for acquiring permission to publish the same. The authors thank Petroliam Nasional Berhad (PETRONAS), Malaysia, for access to the data and Rasidah Husain and Kamal Embong for supporting the project.”.

