# Supplementary material for: The emergence of Miocene reefs in South China Sea and its resilient adaptability under varying eustatic, climatic and oceanographic conditions
Source: Sci Rep. 2020 Apr 28;10:7141. doi: 10.1038/s41598-020-64119-9 (PMC7189246; doi:10.1038/s41598-020-64119-9)
Supplement: Supplementary file 1 — Supplementary information. [file 41598_2020_64119_MOESM1_ESM.pdf]

The emergence of Miocene reefs in South China Sea and its resilient adaptability under varying eustatic, climatic and oceanographic conditions

Manoj Mathew[1,*], Adelya Makhankova[2], David Menier[3], Benjamin Sautter[2], Christian Betzler[4], Bernard Pierson[5]

[1]Shale Gas Research Group, Institute of Hydrocarbon Recovery, Universiti Teknologi PETRONAS, 32610 Bandar Seri Iskandar, Malaysia

[2]Department of Geosciences, Universiti Teknologi PETRONAS, 32610 Bandar Seri Iskandar, Malaysia

[3]Laboratoire Géosciences Océan (LGO), Université Bretagne Sud, 56017 Vannes Cedex, France

[4]Institute of Geology, CEN, University of Hamburg, Bundesstrasse 55, 20146 Hamburg, Germany

[5]GEO-Instituut, Campus Arenberg, Katholieke Universiteit Leuven, Celestijnenlaan 200E, B-3001 Leuven-Heverlee, Belgium

*Corresponding author: Manoj Mathew
Universiti Teknologi PETRONAS
32610 Bandar Seri Iskandar, Malaysia
Phone: +60142361495
Email: manoj_mathew7@yahoo.com

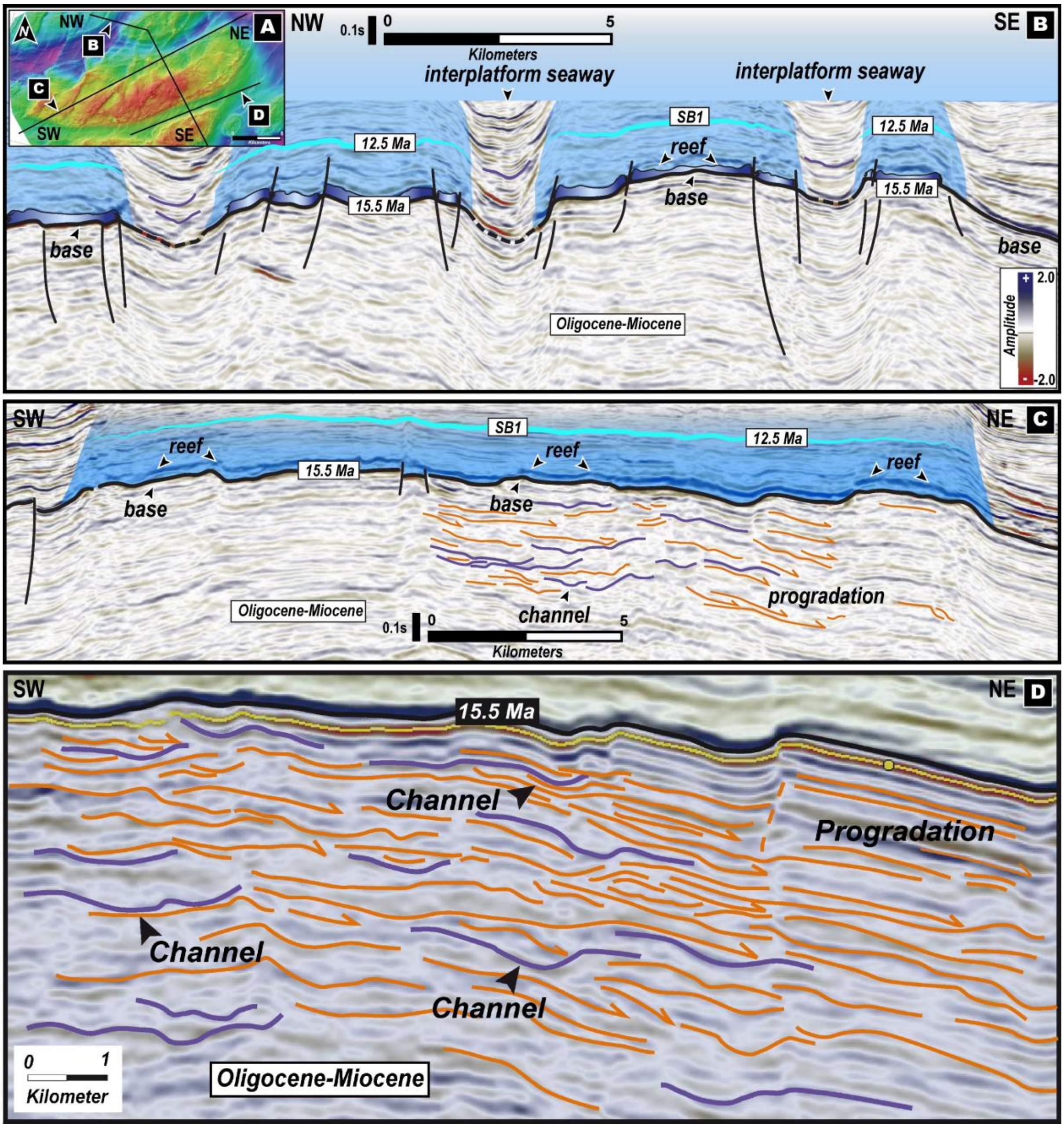

Figure S1. Seismic lines showing Middle Miocene carbonate platforms of Luconia overlying distinct progradation features of Oligo–Miocene paleodelta systems. Interplatform seaways that are confined between the carbonate platforms display an internal architecture that demonstrates repeated incisions by possibly tidal channels and filling phases from above the 15.5 Ma boundary. Below the 15.5 Ma boundary, observations can be made of a sedimentary architecture resembling a progradational deltaic system with numerous channels. Also shown are ages of platform sequence boundaries (SB) recognized and interpreted in this work. Base maps and images in (**A**), (**B**), (**C**) and (**D**) were visualized, interpreted and constructed with Eliis PaleoScan software version 2018.1.0 (http://www.eliis.fr/products/paleoscan%E2%84%A2-software). Figure labels were added using Adobe Illustrator version CS5.1 (http://www.adobe.com/products/illustrator.html).
